# Persistent Neutrophil to Lymphocyte Ratio >3 during Treatment with Enzalutamide and Clinical Outcome in Patients with Castration-Resistant Prostate Cancer

**DOI:** 10.1371/journal.pone.0158952

**Published:** 2016-07-19

**Authors:** Vincenza Conteduca, Simon J. Crabb, Robert J. Jones, Orazio Caffo, Tony Elliott, Emanuela Scarpi, Paolo Fabbri, Lisa Derosa, Francesco Massari, Gianmauro Numico, Sunnya Zarif, Catherine Hanna, Francesca Maines, Helen Joyce, Cristian Lolli, Ugo De Giorgi

**Affiliations:** 1 Istituto Scientifico Romagnolo per lo Studio e la Cura dei Tumori (IRST), IRCCS, Meldola, Italy; 2 Cancer Sciences Unit, University of Southampton, Southampton, United Kingdom; 3 Institute of Cancer Sciences, University of Glasgow, Beatson West of Scotland Cancer Centre, Glasgow, United Kingdom; 4 Medical Oncology Department - Santa Chiara Hospital, Trento, Italy; 5 Christie Hospital, Manchester, United Kingdom; 6 Oncology Unit, Cervesi Hospital, Cattolica, Italy; 7 Medical Oncology Department - Santa Chiara Hospital, Pisa, Italy; 8 Institut Gustave Roussy, Villejuif, France; 9 Medical Oncology and Hematology, Azienda USL della Valle d’Aosta, Aosta, Italy; Carolina Urologic Research Center, UNITED STATES

## Abstract

The baseline value of neutrophil to lymphocyte ratio (NLR) has been found to be prognostic in patients with metastatic castration resistant prostate cancer (CRPC). We evaluated the impact of baseline NLR and its change in patients receiving enzalutamide. We included consecutive metastatic CRPC patients treated with enzalutamide after docetaxel and studies the change of NLR (>3 vs ≤3) after week 4 and 12 weeks. Progression-free survival (PFS), overall survival (OS) and their 95% Confidence Intervals (95% CI) were estimated by the Kaplan-Meier method and compared with the log-rank test. The impact of NLR on PFS and OS was evaluated by Cox regression analyses and on prostate-specific antigen response rates (PSA RR; PSA decline >50%) were evaluated by binary logistic regression. Data collected on 193 patients from 9 centers were evaluated. Median age was 73.1 years (range, 42.8–90.7). The median baseline NLR was 3.2. The median PFS was 3.2 months (95% CI = 2.7–4.2) in patients with baseline NLR >3 and 7.4 months (95% CI = 5.5–9.7) in those with NLR ≤3, *p* < 0.0001. The median OS was 10.4 months (95% CI = 6.5–14.9) in patients with baseline NLR >3 and 16.9 months (95% CI = 11.2–20.9) in those with baseline NLR ≤3, *p* < 0.0001. In multivariate analysis, changes in NLR at 4 weeks were significant predictors of both PFS [hazard ratio (HR) 1.24, 95% confidence interval (95% CI) 1.07–1.42, *p* = 0.003, and OS (HR 1.29, 95% CI 1.10–1.51, *p* = 0.001. A persistent NLR >3 during treatment with enzalutamide seems to have both prognostic and predictive value in CRPC patients.

## Introduction

Over the last decade, an increasing number of novel drugs for castration-resistant prostate cancer (CRPC), including cabazitaxel [1], abiraterone acetate [2, 3], enzalutamide [4, 5], sipuleucel-T [6], and radium-223 [7], have changed the scenario of prostate cancer (PC) management. These new agents have led to longer survival and improved quality of life of these patients. However, the identification of prognostic and predictive factors has also become necessary to help physicians in their choice of treatment and sequencing of drugs. The growing knowledge of tumor biology and/or tumor–host interaction is giving rise to the development of novel circulating biomarkers that facilitate patient risk stratification and the prediction of treatment benefit.

Inflammation, given its important role in the modulation of the cancer microenvironment has emerged over the last few years as a key pathogenic mechanism for carcinogenesis and tumor progression [8–10]. The inflammatory response is detectable in peripheral blood as neutrophilia and/or lymphopenia [9]. Consequently, neutrophil to lymphocyte ratio (NLR) has been proposed as a simple circulating indicator of cancer-related inflammation [10], and has been shown to have prognostic significance in several tumor types [11]. In PC patients, NLR has been evaluated in various disease setting and in association with different drugs, including some new hormonal agents [12–16].

This study assessed the possible predictive and prognostic role of NLR and its modification in CRPC patients treated with enzalutamide, a potent androgen receptor antagonist that inhibits nuclear translocation and DNA binding [17]. Enzalutamide is approved for use in metastatic CRPC patients previously treated with docetaxel [4]. Unlike some other key drugs for PC, enzalutamide is not co-administered with corticosteroids, which may alter NLR due to the accelerated release of neutrophils from the bone marrow into the circulation [18, 19]. We therefore reasoned that studying enzalutamide treated patients would be more informative than studying the correlation between NLR and PC outcome in patients treated with drugs co-administered with steroids, such as docetaxel, cabazitaxel, or abiraterone. In addition, we not only considered NLR value at baseline, but also during treatment, in the hope that it might shed more light on the impact of NLR on CRPC patients treated with enzalutamide.

## Patients and Methods

### Study patients

We retrospectively considered 193 consecutive patients with CRPC treated with enzalutamide after docetaxel between August 2012 and December 2014 in a compassionate-use program in six Italian hospitals (102 patients), or as standard treatment in three UK hospitals (91 patients). The study protocol was approved by the Ethical Committee of Istituto Scientifico Romagnolo per lo Studio e la Cura dei Tumori (I.R.S.T.). Written informed consent was obtained from all patients. Selection criteria included histologically confirmed prostatic adenocarcinoma progressing on androgen deprivation therapy (ADT) and at least 1 prior chemotherapeutic regimen including docetaxel every 3 weeks. Moreover, we considered evaluable for this analysis only patients who received enzalutamide for at least 3–4 weeks. The cutoff values of NLR (>3 vs ≤3) were evaluated at baseline (pre-therapy NLR) and after 4 and 12 weeks (follow-up NLR).

### Treatment and evaluation

Treatment consisted of enzalutamide 160 mg orally daily administered continuously until progressive disease (PD) or interruption for severe toxicity. The Prostate Cancer Clinical Trials Working Group 2 (PCWG2) criteria were used to define response and progression [20]. All recorded PSA test, full blood examinations, including a complete blood count, and scan results were retrospectively collected for these patients, evaluated commonly every 4 weeks for serologic PSA response and as clinically indicated for imaging assessment [21, 22]. In clinical practice a clinical deterioration and/or radiologic evidence of PD was sufficient to establish enzalutamide discontinuation as well as PSA increase associated with therapy interruption secondary to unacceptable toxicity or death.

### Statistical Analysis

Data were summarized by frequency for categorical variables and by median and range for continuous variables. As baseline PSA value, pre-therapy NLR value, NLR 4 weeks and baseline LDH were not normal distributed, they were log transformed.

Association between categorical variables was assessed using the Fisher’s exact test, when appropriate. Progression-free survival (PFS) was calculated from the start of therapy with enzalutamide until disease progression or last follow-up. Patients who died without progression were censored at the time of death. Overall survival (OS) was calculated from the start of therapy until death. Patients lost to follow-up were censored at the time of last contact. The Kaplan–Meier method was used to estimate PFS and OS. The Cox proportional hazard regression were used to compare survival between groups of patients on the basis of the NLR ratio. After univariate analysis, a multivariate analysis was carried out by Cox regression model and included the following variables:

- Eastern Cooperative Oncology Group (ECOG) performance status,
- visceral disease including lung, liver, brain, and bone metastases (present vs. absent),
- baseline PSA value,
- pre-therapy NLR value,
- change in NLR.

In multivariate analysis, we compared Bayesian Information Criterion (BIC) for a model with and a model without the pre-therapy NLR. As BIC for the model without pre-therapy NLR was lower than the model with pre-therapy NLR, it was dropped from the final model. Patients who did not survive 4 weeks (or 12 weeks) were excluded from the analysis related to change in NLR. Differences were considered statistically significant when P<0.05. All statistical analyses were carried out with SAS statistical software, version 9.3 (SAS Institute, Cary, NC).

## Results

### Patient characteristics

This study included 193 consecutive CRPC patients from 9 centers. Median age was 73.1 years (range 42.8–90.7). Median follow-up was 11.7 months (range 0.5–27.4). The median baseline NLR was 3.2. One hundred and five patients (54.4%) had baseline NLR >3. At study entry, 58 patients (30.1%) had visceral metastases (including liver, lung, bone, and brain sites) and 112 (58.3%) had multiple metastatic sites. Sixty-seven patients (35.1%) had received two or more therapeutic lines. Baseline characteristics did not significantly differ according to baseline NLR<3 or ≥3 (S1 Table).

### NLR and clinical outcome

At the time of analysis, 149 (77.2%)of the193 patients had progressive disease, and 106 (54.9%) had died. The median PFS was 4.8 months (95% CI 3.7–6.0), and the median OS was 13.9 months (95% CI 10.1–15.9). In relation to NLR value, the median PFS was 7.4 months (95% CI 5.5–9.7) in patients with pre-therapy NLR ≤3 and 3.2 months (95% CI 2.7–4.2) in those with pre-therapy NLR >3 (*p* < 0.0001). The NLR was also associated with difference in PFS if it was assessed after treatment had started. After 4 weeks, the median PFS was 6.2 months (95% CI 4.7–9.0) in 113 patients with NLR ≤3and 3.0 months (95% CI 2.6–4.2) in 71 with NLR>3 (*p* < 0.0001). After 12 weeks, the median PFS was 9.3 months (95% CI 5.4–10.1) in 77 patients with NLR ≤3 and 3.4 months (95% CI 2.7–4.8) in 45 with NLR >3 (*p* < 0.0001).

The median OS was 16.9 months (95% CI 11.2–20.9) and 10.4 months (95% CI 6.5–14.9) (*p* < 0.0001) in patients with pre-therapy NLR ≤3 or >3, respectively. After 4 weeks median OS was 15.9 months (95% CI 11.7–20.9) in the NLR group ≤3 and 7.1 months (95% CI 6.4–10.4) (*p* < 0.0001) in those with NLR >3 (*p* < 0.0001). At 12 weeks, patients with NLR ≤3 showed a median OS of 20.2 months (95% CI 15.1–21.0) compared to 8.2 months (95% CI 6.2–14.9) (*p* = 0.002) of the NLR >3 group.

**Fig 1:**
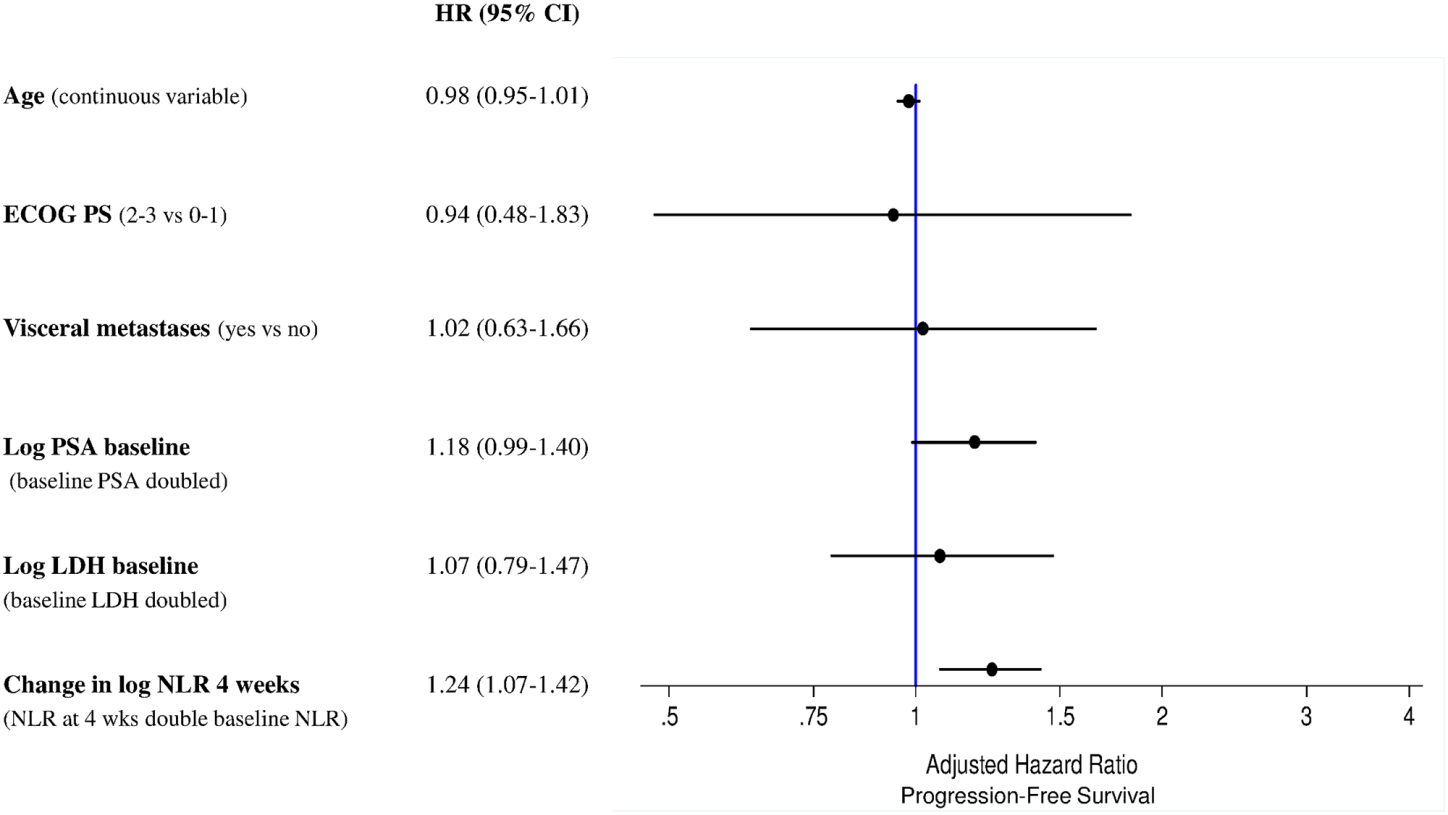
Forest plot for progression-free survival.

**Fig 2:**
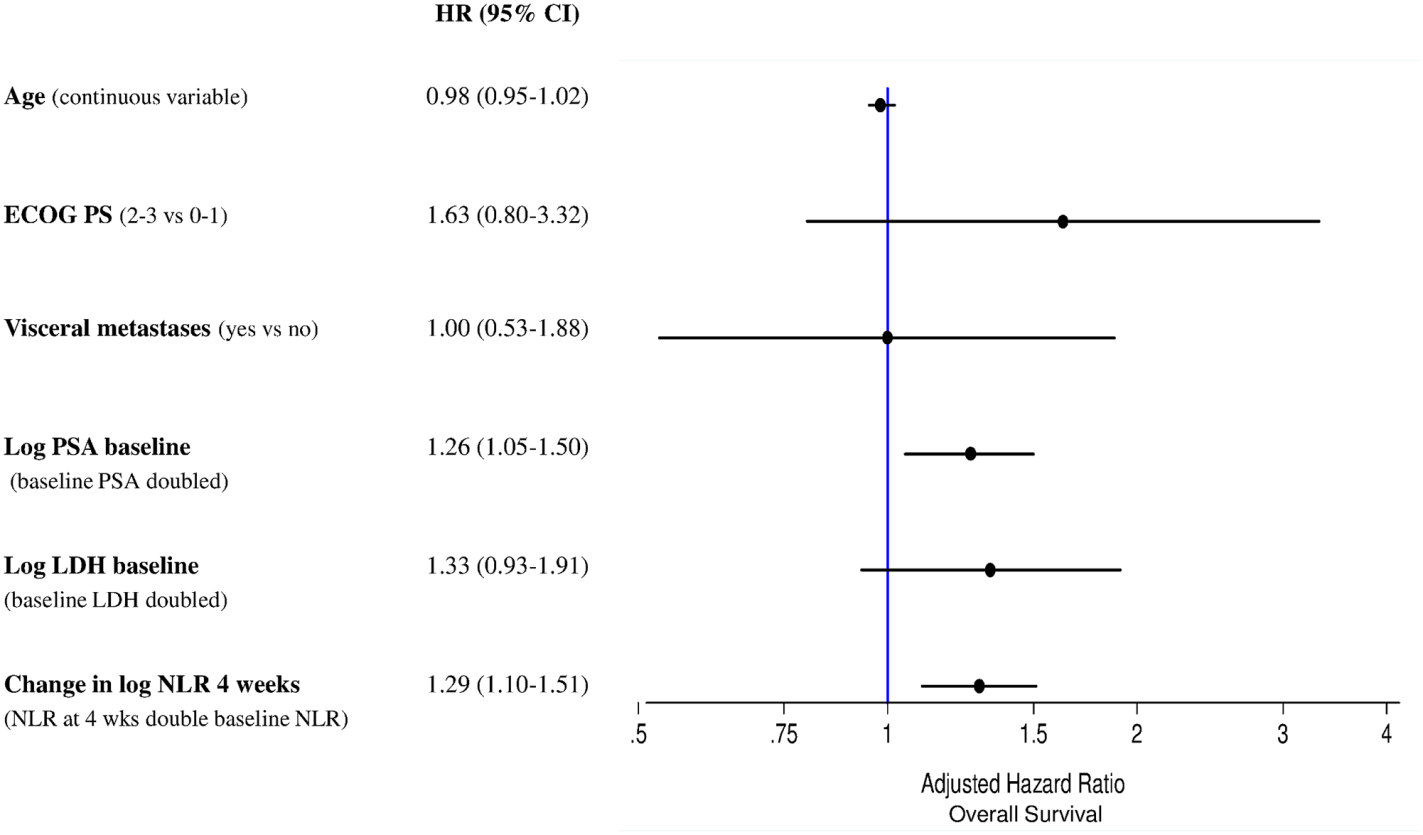
Forest plot for overall survival.

A multivariate analysis revealed that follow-up NLR after 4 and 12 weeks’ treatment with enzalutamide was a significant predictor of PFS with HR 1.24 (95% CI 1.07–1.42) (*p* = 0.003) and HR 1.09 (95% CI 1.01–1.19) (*p* = 0.031), respectively. Similar results were also observed for OS for NLR after 4 weeks: HR 1.29 (95% CI 1.10–1.51) (*p* = 0.001), whereas were of borderline significance for NLR after 12 weeks: HR 1.09 (95% CI 0.99–1.20) (*p* = 0.079). Forest plots for PFS and OS, are presented in Figs 1 and 2, respectively. Therneau-Gramschs test failed to find, for PFS and OS analyses, violations of the PH assumption.

### Changes in NLR and PSA response rate

We divided up the two pre-therapy NLR groups (NLR ≤3 or >3) on the basis of the follow-up NLR (≤3 or > 3), obtaining 4 subgroups: 1) low-low (pre-therapy NLR ≤3 and follow-up NLR ≤3); 2) low-high (pre-therapy NLR ≤3 and follow-up NLR >3); 3) high–low (pre-therapy NLR > 3 and follow-up NLR ≤3); and 4) high–high (pre-therapy NLR > 3 and follow- up NLR > 3).

Changes in NLR were correlated with PSA response rate. Tables 1 and 2 show changes in NLR and PSA response rate at 4 and 12 weeks, respectively. The groups of patients with high-low NLR and high-high NLR were associated with poorer PSA response rate either at 4 weeks or 12 weeks than the group with low-low NLR. PSA response rate could be considered as a mirror of the association between NLR change and clinical outcome (Tables 1 and 2).

**Table 1:** Changes in NLR and PSA response rate at 12weeks.

PSA RR	<50%	>50%			
	No. pts (%)	No. pts (%)	*p*^1^	OR (95% CI)^2^	*p*^2^
**Low-Low**	18 (29.5)	40 (65.6)		1.00	
**Low-High**	7 (11.5)	3 (4.9)		0.32 (0.07–1.44)	
**High-Low**	11 (18.0)	8 (13.1)		0.47 (0.14–1.54)	
**High-High**	25 (41.0)	10 (16.4)	0.0002	0.66 (0.25–1.72)	0.364

Abbreviations: PSA, prostate-specific antigen; RR, response rate; pts, patients; NLR, neutrophil to lymphocyte ratio.

^1^unadjusted;

^2^adjusted by age, ECOG PS, visceral metastases, Log PSA baseline, Log LDH baseline

**Table 2:** Changes in NLR and PSA response rate at 4 weeks.

PSA RR	<50%	>50%			
	No. pts (%)	No. pts (%)	*p*^1^	OR (95% CI)^2^	*p*^2^
**Low-Low**	25 (30.1)	52 (57.1)		1.00	
**Low-High**	7 (8.4)	3 (3.3)		0.16 (0.04–0.73)	
**High-Low**	16 (19.3)	20 (22.0)		0.60 (0.26–1.42)	
**High-High**	45 (54.2)	16 (17.6)	<0.0001	0.27 (0.13–0.58)	0.003

Abbreviations: PSA, prostate-specific antigen; RR, response rate; pts, patients; NLR, neutrophil to lymphocyte ratio.

^1^unadjusted;

^2^adjusted by age, ECOG PS, visceral metastases, Log PSA baseline, Log LDH baseline

### Changes in NLR and survival

**Fig 3:**
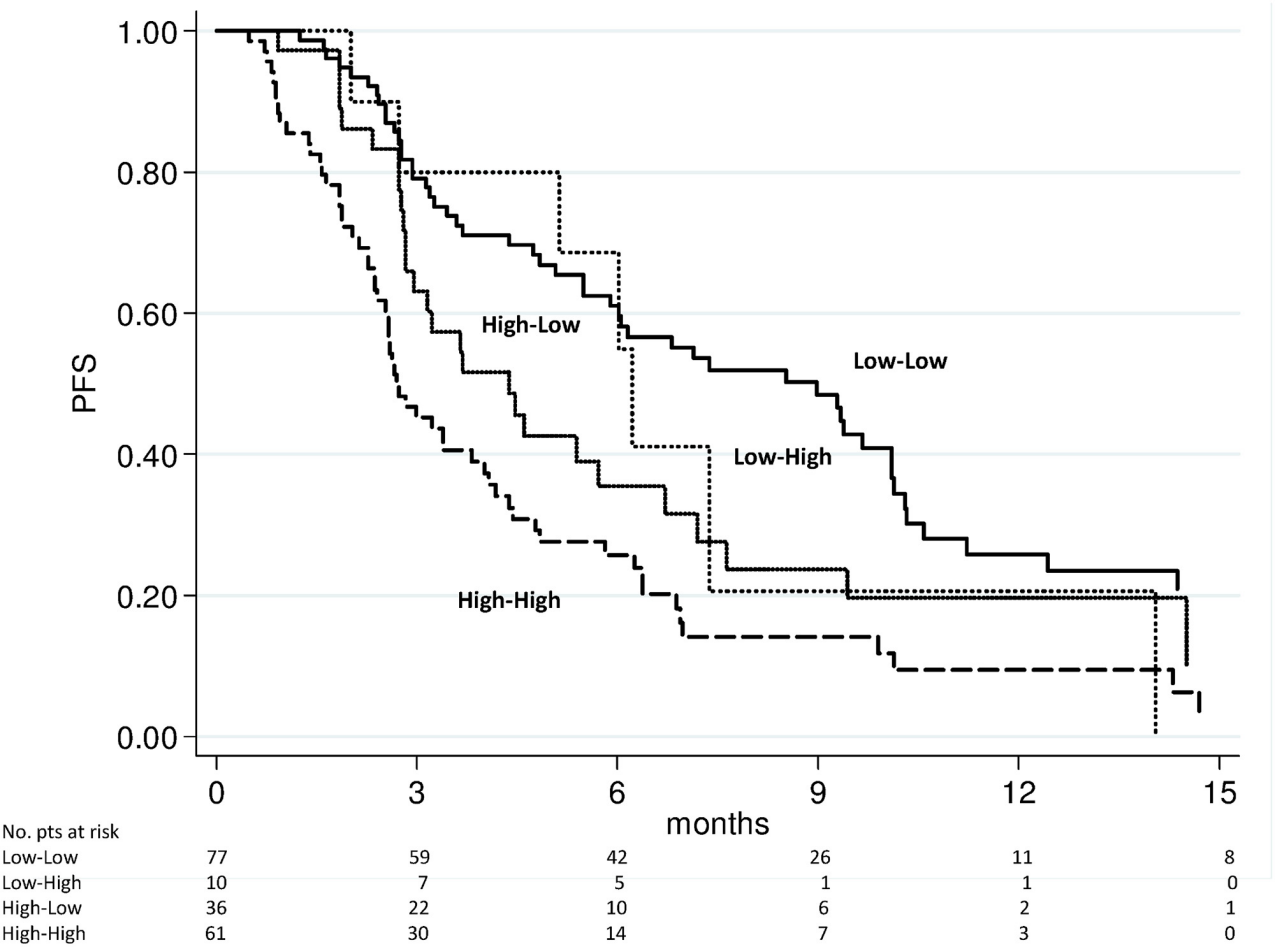
Progression-free survival according to pre-therapy and follow-up NLR at 4 weeks of enzalutamide treatment.

**Fig 4:**
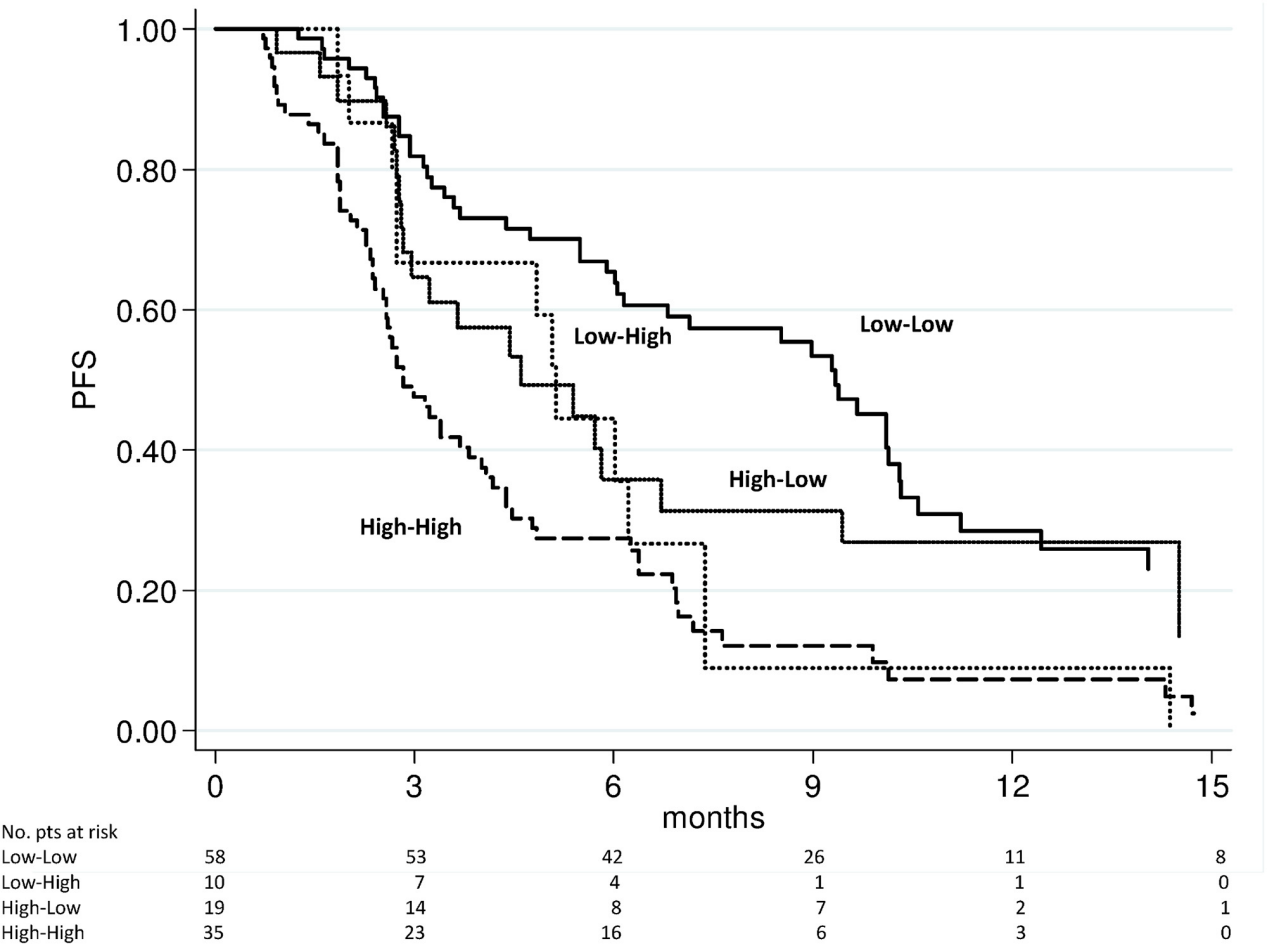
Progression-free survival according to pre-therapy and follow-up NLR at 12 weeks of enzalutamide treatment.

**Fig 5:**
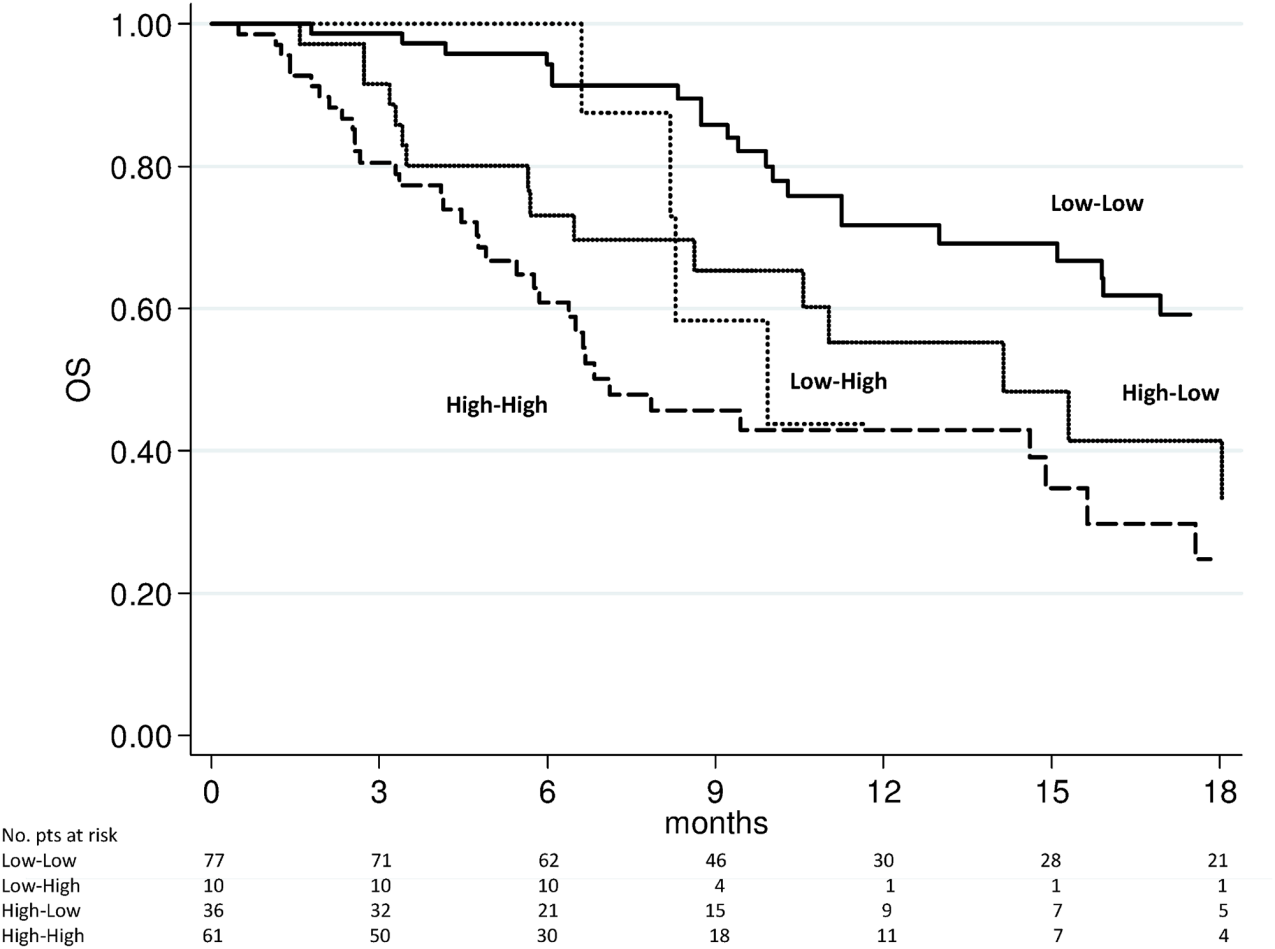
Overall survival according to pre-therapy and follow-up NLR at 4 weeks of enzalutamide treatment.

**Fig 6:**
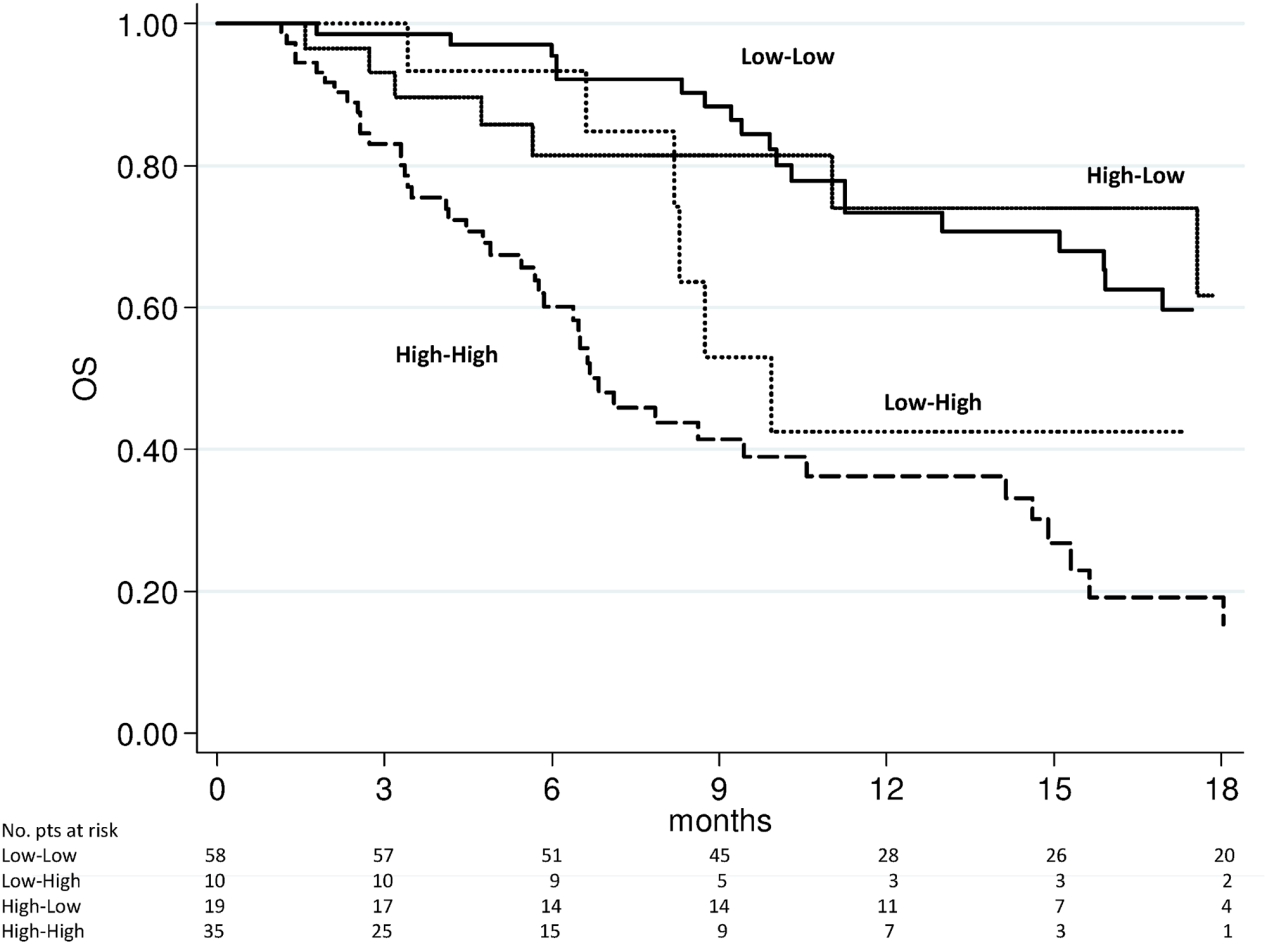
Overall survival according to pre-therapy and follow-up NLR at 12 weeks of enzalutamide treatment.

We evaluated the above-mentioned 4 subgroups for PFS and OS. The high-high group showed a significantly worse PFS (Figs 3 and 4) and OS (Figs 5 and 6) after 4 weeks of treatment with enzalutamide [HR 2.56 (95% CI 1.75–3.73) (*p* < 0.0001) and HR 2.87 (95% CI 1.82–4.53) (*p* < 0.0001), respectively] and this was further confirmed in the NLR analysis performed after 12 weeks [HR 2.85 (95% CI 1.94–4.20) (*p* < 0.0001) and HR 3.02 (95% C1.93–4.72) (*p* < 0.0001), respectively]. The high-low group characterized by a significantly poorer OS at 4 weeks [HR 1.95 (95% CI 1.12–3.40) (*p* = 0.018)] than the low-low group, while patients with low-high NLR had a worse PFS after 12 weeks than low-low group [HR 2.04 (95% CI 1.10–3.80) (*p* = 0.024)] (Table 3). A doubling of the NLR at 4 weeks, compared with baseline NLR, increased the hazard of death/recurrence by about 25%, adjusted for all other factors.

**Table 3:** Change in NLR and survival.

		**PFS**				**OS**			
**4 weeks**	**n. pts**	**n. events**	**Median value (months) (95% CI)**	**HR (95% CI)**	***p***	**n. events**	**Median value (months) (95% CI)**	**HR (95% CI)**	***p***
**Low-Low**	77	56	9.0 (5.9–10.1)	1.00		39	20.2 (13.0–21.0)	1.00	
**Low-High**	10	7	6.2 (2.0–14.0)	1.32 (0.60–2.92)	0.491	5	9.9 (6.2-nr)	1.75 (0.68–4.51)	0.249
**High-Low**	36	26	4.4 (2.8–6.7)	1.50 (0.94–2.38)	0.085	19	13.9 (6.5–17.2)	1.95 (1.12–3.40)	0.018
**High-High**	61	51	2.7 (2.4–4.0)	2.56 (1.75–3.73)	<0.0001	36	6.8 (5.8–14.6)	2.87 (1.82–4.53)	<0.0001
		**PFS**				**OS**			
**12 weeks**	**n. pts**	**n. events**	**Median value (months) (95% CI)**	**HR (95% CI)**	***p***	**n. events**	**Median value (months) (95% CI)**	**HR (95% CI)**	***p***
**Low-Low**	58	38	9.3 (6.1–10.3)	1.00		29	20.2 (13.0–21.0)	1.00	
**Low-High**	10	8	5.1 (2.7–7.4)	2.04 (1.10–3.80)	0.024	4	8.7 (6.6-nr)	0.99 (0.43–2.25)	0.980
**High-Low**	19	11	4.6 (2.8–6.7)	1.38 (0.82–2.32)	0.229	4	17.2 (10.4–21.0)	1.18 (0.61–2.29)	0.613
**High-High**	35	27	2.8 (2.5–4.0)	2.85 (1.94–4.20)	<0.0001	21	6.7 (5.7–10.6)	3.02 (1.93–4.72)	<0.0001

Abbreviations: NLR, neutrophil to lymphocyte ratio; pts, patients; PFS, progression free survival; OS, overall survival; HR; hazard ratio; CI, confidence interval; nr, not reached.

## Discussion

The present study evaluated NLR as a potential biomarker in men with CRPC treated with enzalutamide, and confirmed the role of inflammation in prostate carcinogenesis, disease progression, CRPC development and resistance to treatment [22]. In recent years, numerous *in vitro* and *in vivo* studies have suggested that there may be crosstalk among tumor cells, the microenvironment and inflammatory cells in PC have suggested a cross-talk among tumor cells, the microenvironment and inflammatory cells in PC [23–25]. The involvement of the host’s immune system in all stages of PC has been confirmed by the increased production of some cytokines including interleukin (IL)-1, IL-4 and IL-6, macrophage inhibitory cytokine 1 [26–29] and, recently, by the altered expression of modulator genes of the immune system [30]. Ross et al.’s prospective study developed a whole-blood RNA transcript-based prognostic model of CRPC patients [30] that included upregulated genes (*C1QA*, *TIMP1* and *CDKN1A*) known to be involved in the differentiation of monocytes to macrophages and downregulated genes (*ABL2*, *ITGAL* and *SEMA4D*) that control T-cell motility and activity. Systemic inflammation, fibrinogen, IL-6, and C-reactive protein levels have also been hypothesized as prognostic and predictive biomarkers in PC, albeit with conflicting results [31–33]. Furthermore, the platelet to lymphocyte ratio (PLR) been also assessed in PC patients as biomarkers of outcome and resistance to therapy [12–16, 34].

NLR is believed to reflect the equilibrium between innate (neutrophil) and adaptive (lymphocyte) immune response. Its association with higher serum PSA levels observed in our study may indicate an impairment of the adaptive host’s capacity to regulate inflammation. We used 3 as a threshold for NLR, classifying patients with NLR >3 as having a ‘high’ ratio and those with NLR ≤3 as having a ‘low’ ratio. Using this cut-off we confirmed the strong association between NLR and PSA response, previously reported in other studies in PC patients treated with cabazitaxel and abiraterone [15, 16].

The majority of previous studies focused only on pre-therapy NLR values in PC patients, whereas we also assessed the prognostic impact of NLR during treatment. A change in NLR may reflect the dynamic alterations of balance among host inflammatory response, immune response, and the effect of treatment. The results of our study were not compromised by the problem of data interpretation relating to the concomitant use of corticosteroids, which are used in combination with abiraterone. Then, our results, in terms of prediction, can only be applied to studies similar to this one, but not to those with concomitant use of corticosteroids. Steroids often alter NLR value by reducing the number of lymphocytes, redistributing them in other body compartments (e.g. spleen, lymph nodes, and bone marrow) and accelerating the release of neutrophils from the bone marrow into the circulation. Consequently, patients on corticosteroids usually have a higher NLR, as reported by Lorente *et al*. [15] who demonstrated that NLR prognostic value was independent of the prior use of corticosteroids.

Our study revealed the importance of evaluating NLR values before and during treatment because patients in the high-high risk group (pre-therapy and follow-up NLR > 3) had a markedly worse prognosis, (Figs 3 to 6), with results of the multivariate analysis that suggest NLR after 4 weeks of enzalutamide as better predictor of OS (HR = 1.29, *p* = 0.001) than NLR after 12 weeks (HR = 1.09, *p* = 0.079) (Table 3). However, when dividing patients for subgroups according to baseline and follow-up NLR, a persistent NLR >3 was able to strongly predict the clinical outcome after either 4 or 12 weeks (Table 3, Figs 3 to 6).

Although follow-up NLR has been investigated in other urological malignancies as a relevant prognostic factor [35, 36], we analyzed NLR at two different treatment times, with comparable results. In conclusion, the results from the present study would seem to indicate that a persistent increase in NLR during enzalutamide has both a prognostic and predictive value in patients with CRPC. Currently, the identification of non-invasive markers in PC patients treated with new hormonal drugs, such as enzalutamide, is the object of much of the translational research carried out into this area, and NLR could, like PSA, prove to be an important factor to monitor treatment in PC patients. It must, however, be pointed out that our work is somewhat limited by the retrospective nature of the analysis, the sample size, the absence of external validation, and the heterogeneity of previous lines of therapy administered. An independent validation of our findings is thus needed in a larger prospective study.

## Supporting Information

S1 Table Clinical Data Neutrophil to lymphocyte ratio and Enzalutamide Final. (XLS)

## Abbreviations

ADT: androgen deprivation therapy
BIC: Bayesian Information Criterion
CI: confidence interval
CRPC: castration-resistant prostate cancer
CT: computed tomography
CTCAE: Common Terminology Criteria for Adverse Events
DNA: Deoxyribonucleic acid
ECOG: Eastern Cooperative Oncology Group
HR: hazard ratio
LDH: lactate dehydrogenase
NLR: neutrophil to lymphocyte ratio; nr, not reached
PSA: prostate-specific antigen
OS: overall survival
PC: prostate cancer
PFS: progression free survival
PCWG: Prostate Cancer Working Group
PD: progressive disease
PSA: Prostate-Specific Antigen
RECIST: Response Evaluation Criteria in Solid Tumors
RR: response rate
